# Evidence on the need for an integrated approach to the management of diabetes: the surgical perspective

**Published:** 2020-02-20

**Authors:** C Setacci, G de Donato, M Tadiello, M Tozzi, G Bracale, L del Guercio, UM Bracale, F Setacci

**Affiliations:** 1 Vascular Surgery Unit, University of Siena, Italy; 2 Vascular Surgery Unit Insubria, University of Varese, Italy; 3 Vascular Surgery Unit, Department of Public Health, University Federico II of Naples, Naples, Italy; 4 Vascular Surgery Unit IRCCS Multimedia, Milano, Italy

## I. EDITORIAL

History of the team approach to management of the diabetic foot chronicles the emergence of the specialties of vascular surgery and wound healing. The partnership between the diabetologist, vascular surgeon, and podiatrist can be seen as a natural marriage that complements the skills and knowledge of each partner and results in more successful limb salvage and functional outcomes^1^.

Diabetic foot patients are among the most complex and vulnerable of all diabetes patients, with high morbidity and mortality. Specialized diabetic foot clinics of the 21st century should be equipped to coordinate revascularization procedures, to aggressively treat infections, and to manage medical comorbidities within a multidisciplinary forum.

History has taught us that optimal management of diabetic foot complications is best provided in a hospital-based diabetic foot clinic^2^. It is common experience that clinic must be available to manage emergencies and equipped to perform urgent investigations, wound debridement, and to initiate immediate parenteral antibiotic therapy. It must also be able to obtain rapid vascular, podiatric, and orthopaedic opinions and to arrange for emergency admissions to the hospital

However, aside daily experience do we have evidences that multidisciplinary team (MDT) has an impact in the management of individuals with diabetic foot?

In 2017, Buggy A. et al tried to explore the delivery of a diabetic foot MDT in detail and to identify if its existence leads to more positive diabetic foot outcomes^3^. Thus, their systematic review explored the following question: What is the impact of the MDT in the management of individuals with DFUs?

Their review counted 19 eligible studies in which the impact of the multidisciplinary team in the management of the diabetic foot compared with those who did not receive multidisciplinary care was assessed. They found some positive effects of the MDT on DFUs, namely; amputation rate, severity of amputation and resource use. MDT care also appears to improve mortality and quality of life of people with this condition.

MDTs was heterogeneous; they were constituted for the most by diabetologist-endocrinologists, vascular-general surgeon and/or orthopaedics, podiatrists and nurses^4^. Even if it is impossible to perform a correct statistical analysis because of the high heterogeneity of data and results, it is notable that MDTs where Vascular Surgeon was absent had the highest major amputation rates (9, 5–47%, mean 34%) compared to the groups where Vascular Surgeon was included (4–19%, mean 12, 5%).

That data is without statistical weight but it is the mirror of what we live daily; in our daily practice we are used to see frequently diabetic patients with terrific diabetic foot gangrene sent by small peripheral centres where the Vascular Surgeon is not available. That is the reason why we strongly recommend an integrated, coordinate, multidisciplinary care.

To date, in literature, different combinations of team members are described (in absence of comparisons among different team compositions) and some studies do not discuss how the team was formed. However we believe that a foot care team would consist ideally of a diabetologist, a vascular surgeon, an, a specialised nurse, a podiatrist, an educator and a plaster technician. In particular, it would be advisable that the multidisciplinary team leader for diabetic foot care would be a diabetologist because diabetic foot is a chronic complication of diabetes and poor vascular supply due to arterial disease involving small as well as large vessels is strongly influenced by the underlying pathology^5^.

In addition, current guidelines recommend the involvement of a multi-disciplinary team for the treatment of diabetic foot ulcers. The International Working Group on the Diabetic Foot (IWGDF) guidance documents on prevention and management of foot problems in diabetes published in 2015 suggested: (1) offloading and protection of the ulcer, (2) perfusion of the foot, (3) antibiotic therapy for infection treatment, (4) metabolic control and treatment of comorbidity, (5) local wound care, (6) education for patient and relatives and (7) prevention of recurrence^6^.

American Diabetes Association too recommends a multidisciplinary approach for individuals with foot ulcers and high-risk feet. In particular, the presence of a multidisciplinary team aimed at preventive care has been reported to decrease the risks associated with diabetic foot and amputation by 50% to 85%^7^,^8^.

National Institute for Health and Care Excellence has developed guidelines on the prevention and management of the diabetic foot^9^. The need for a person with diabetes to be referred to a multidisciplinary team is repeated throughout these guidelines, at all stages of disease progression and for its prevention. Even if these guidelines agree that there is no unanimous vision about the professionals who are the key components of the multidisciplinary team it should consist of health care professionals who have the resources and specialist skills. It should normally include a diabetologist, a surgeon with relevant expertise in managing diabetic foot problems (vascular and orthopaedic surgeons), a microbiologist, an interventional radiologist, a diabetes nurse specialist, a podiatrist and a tissue viability nurse, together with the access to other specialist services required (such as rehabilitation services, plastic surgery, psychological services and nutritional services).

The Società Italiana di Diabetologia and the Associazione Medici Diabetologi to ensure homogeneous and appropriate care for all patients with foot injuries have also proposed an organization of diabetic structures based on three levels of complexity^10^. In guidelines published in 2018, drawn up by these two leading Italian scientific diabetes societies, it was reiterated that the prevention and care team for diabetic foot problems should include experienced clinicians, but also a staff with educational skills and trained for diabetic foot care(e.g. podiatrists and nurses)

A number of diabetes-related health policy guidelines are present in Italy, providing indications about general practitioner, primary care and hospital teaching specialist assessment in the three levels of complexity discussed, according to the international scientific guidelines. This organizational model represents the best way to manage diabetic foot ulcers, if adapted to the various health care realities, as well as to the various economic resources. The multidisciplinary approach and the creation of a shared clinical pathway allowed the institution of the so-called “pathway of diagnosis therapy and assistance” for diabetic foot problems. In 2011, the Italian Ministry of Health cited the pathway of diagnosis therapy and assistance introduced at Siena University Hospital as a good example of diabetic foot multidisciplinary team^11^.

In this pathway we suggested a protocol that is still our gold standard, but it is in constant develop due to materials and technical advancement^12^.

1. Early diagnosis with a 24 h on call DF team. All the members of the team should be able to perform a duplex scan and to identify an infective disease, if present.
2. Urgent treatment of severe foot infection with an aggressive surgical debridement.
3. Early revascularization within 24 hours. In all cases, the first line approach should be represented by endovascular procedures (PTA ± stenting)^13^.
4. Definitive treatment: wound healing, reconstructive surgery, and orthesis.

This solution is also recommended by the most recent guidelines, in particular by International Guidelines on the treatment of diabetic foot and the Guidelines of the European Society of Vascular and Endovascular Surgery of critical limb ischemia and diabetic foot

Therefore, Vascular Surgeon cannot act alone, and he must use all arrows in his bow, more and more in recent years when new advances in material and technique are sprouting. Techniques such as subintimal angioplasty of the femoral-popliteal artery segment, retrograde angioplasty using transpedal access, arterial flossing with anterograde-retrograde intervention, drug-coated balloon and stent angioplasty, and transcollateral angioplasty are several technical innovations that improve the percutaneous transluminal angioplasty success rate in the diabetic limb. We can also count on new cellular therapies such as on-site harvesting and injection of autologous monocells for help wound healing after revascularisation in extreme situations, or in the worst cases for non-revascularisable patients^14^.

The battle with diabetes has to be conducted by all the Team and we have weapons for doing it.
